# Regulation of Travel and Traffic

**Published:** 1890-05

**Authors:** 


### Regulation of Travel and Traffic, Pennsylvania State Board of Health.

This is a pamphlet which gives the rules permitting the transportation of dead bodies from one place to another, with directions how to get a permit, etc.

